# Supplementary material for: Gene regulatory network structure informs the distribution of perturbation effects
Source: bioRxiv. 2024 Oct 22:2024.07.04.602130. Originally published 2024 Jul 5. Preprint. [Version 2] doi: 10.1101/2024.07.04.602130 (PMC11245109; doi:10.1101/2024.07.04.602130)
Supplement: Supplement 2 [file NIHPP2024.07.04.602130v2-supplement-2.pdf]

bioRxiv preprint doi: https://doi.org/10.1101/2024.07.04.602130; this version posted October 22, 2024. The copyright holder for this preprint (which was not certified by peer review) is the author/funder, who has granted bioRxiv a license to display the preprint in perpetuity. It is made available under aCC-BY 4.0 International license.

844 # Supplementary Information

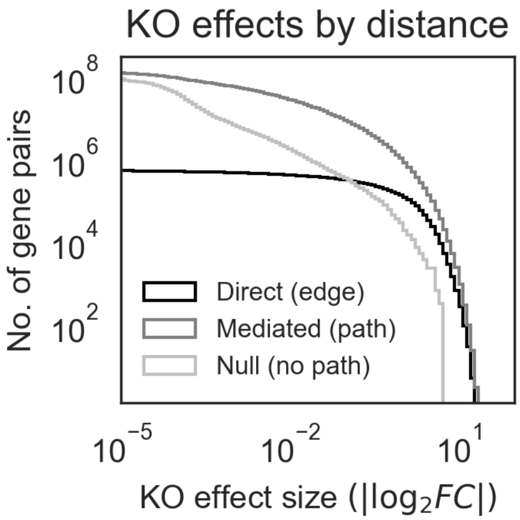

Figure S1: Mediated effects outnumber direct effects at most magnitudes. Same as Fig. 3D, but with distances binned by whether pairs of genes are connected by an edge (distance 1, a "direct effect"), any path (distance greater than 1, a "mediated effect"), or no path at all ("null"). Note also that the y-axis is the count of gene pairs with a perturbation effect of at least the magnitude given on the x-axis – that is, the distribution shown is a non-normalized inverse CDF. Gene pairs are pooled from the 50 example GRNs in Fig. 3.

bioRxiv preprint doi: https://doi.org/10.1101/2024.07.04.602130; this version posted October 22, 2024. The copyright holder for this preprint (which was not certified by peer review) is the author/funder, who has granted bioRxiv a license to display the preprint in perpetuity. It is made available under aCC-BY 4.0 International license.

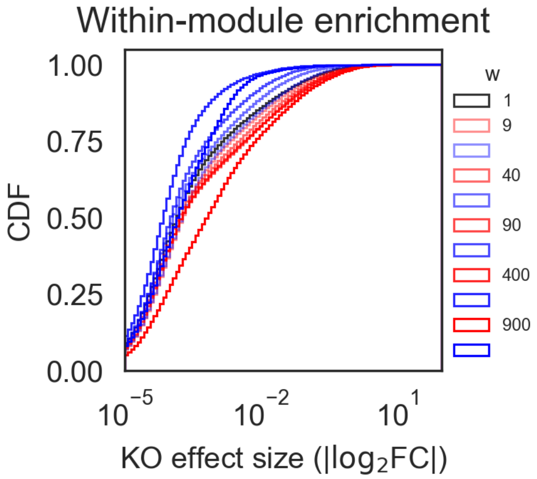

Figure S2: **Modularity term differentiates within- and between-module effects**. Same as Fig. 3E, with within-module perturbation effects in red and between-module perturbation effects in blue. Here, networks are chosen so as to highlight the effect of the modularity term w. Each pair of blue and red tracelines is distribution of the within- (red) or across-module perturbation effects a single GRN. The generating parameters for these GRNs vary w (see legend) but hold other parameters constant, as follow: $p = 1/4$, $k = 50$, $\delta_{in} = 10$, $\delta_{out} = 10$.

bioRxiv preprint doi: https://doi.org/10.1101/2024.07.04.602130; this version posted October 22, 2024. The copyright holder for this preprint (which was not certified by peer review) is the author/funder, who has granted bioRxiv a license to display the preprint in perpetuity. It is made available under aCC-BY 4.0 International license.

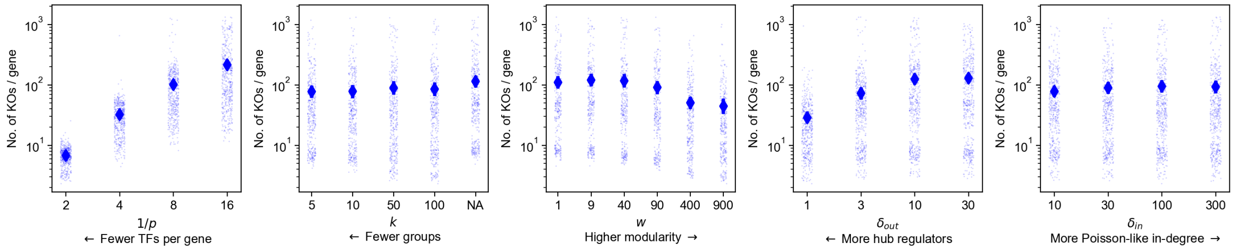

Figure S3: **Network generating parameters affect the number of KO effects**. Same as Fig. 4, but with summary statistic (y-axis) as the average number of perturbation effects per gene in the GRN with $|\log_2 \mathrm{FC}| \geq 0.1$. We observe a similar direction of effect for each parameter as with the statistics presented in the main text.

bioRxiv preprint doi: https://doi.org/10.1101/2024.07.04.602130; this version posted October 22, 2024. The copyright holder for this preprint (which was not certified by peer review) is the author/funder, who has granted bioRxiv a license to display the preprint in perpetuity. It is made available under aCC-BY 4.0 International license.

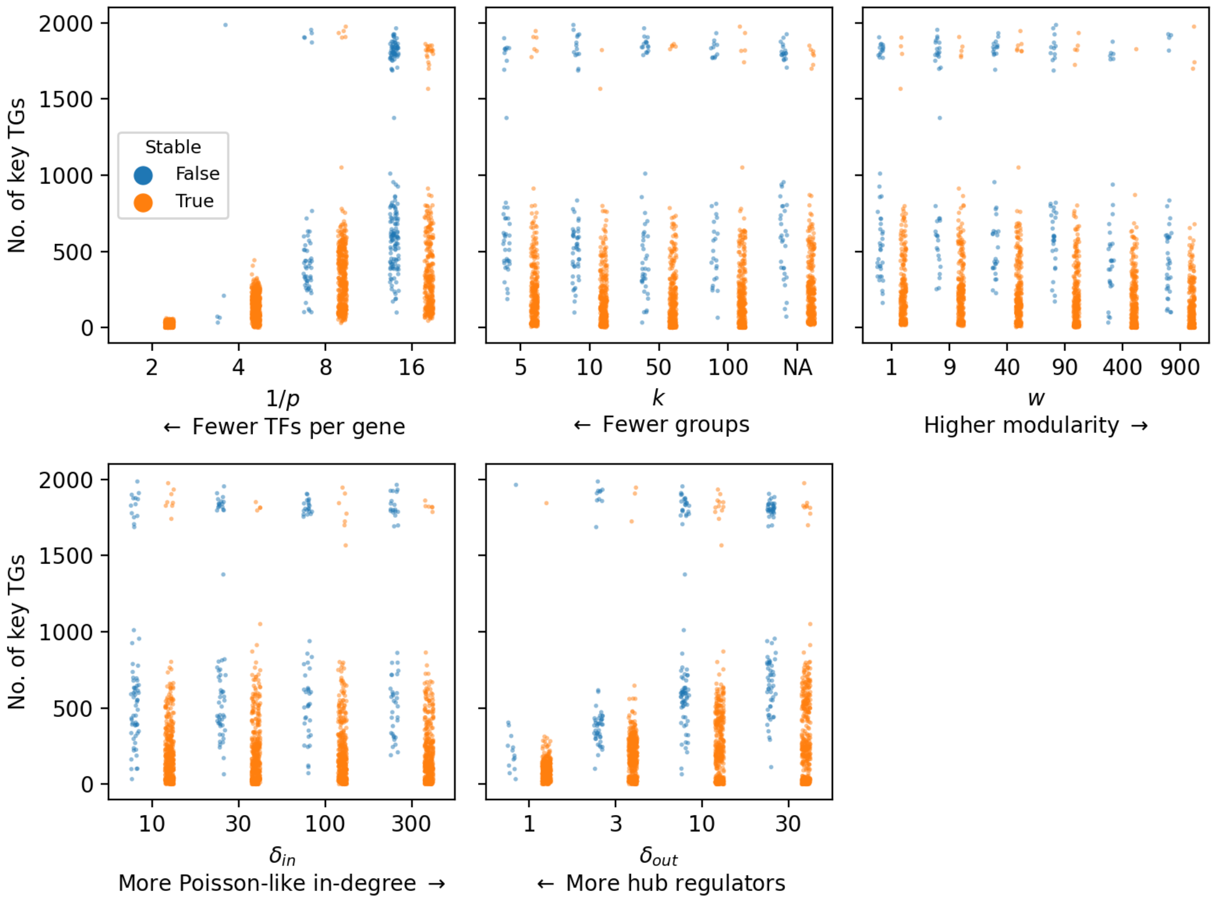

Figure S4: Network generating parameters affect the stability of the fixed point. Same as Fig. 4, only showing the number of key target genes and stratifying by whether the expression equilibrium point of the synthetic GRN is stable (**Methods**). In all, 1,693 of the 1,920 GRNs (88.2%) reach an expression equilibrium through forward simulation of the SDE which is a stable fixed point of the corresponding ODE. These GRNs tend to be sparse (lower $1/p$), modular (higher w), and have more hub regulators (lower δ_{out}).

bioRxiv preprint doi: https://doi.org/10.1101/2024.07.04.602130; this version posted October 22, 2024. The copyright holder for this preprint (which was not certified by peer review) is the author/funder, who has granted bioRxiv a license to display the preprint in perpetuity. It is made available under aCC-BY 4.0 International license.

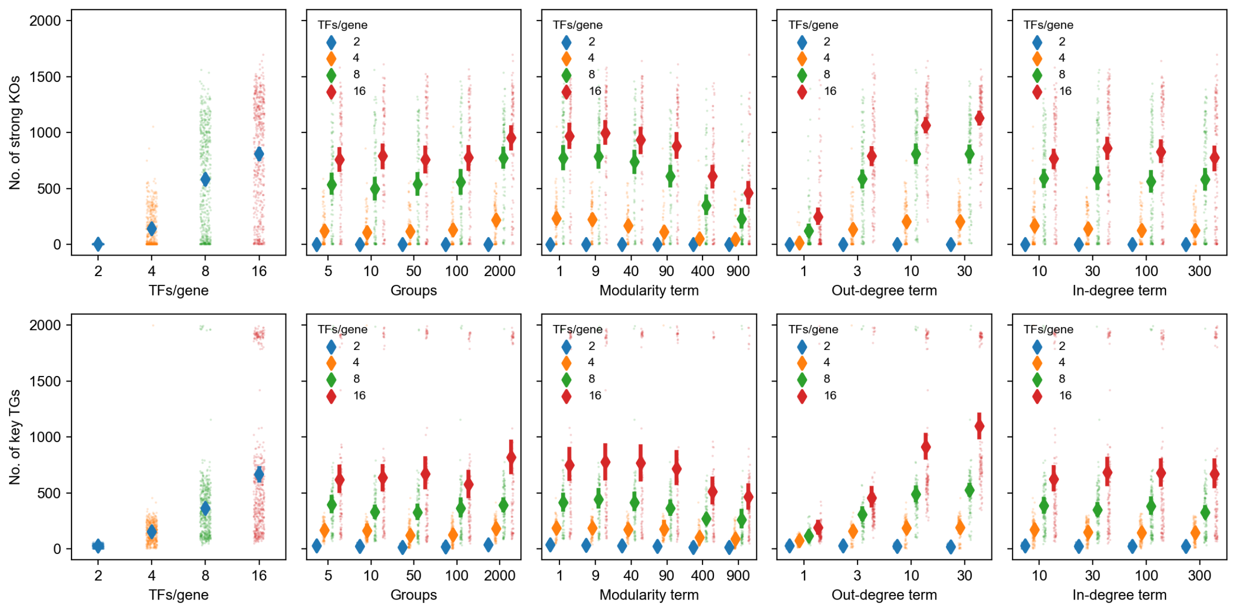

Figure S5: No interaction between sparsity term and other network generating parameters. Same as Fig. 4, but with additional stratification by the sparsity term $1/p$. There is no obvious visual evidence for interactions between the parameters.

bioRxiv preprint doi: https://doi.org/10.1101/2024.07.04.602130; this version posted October 22, 2024. The copyright holder for this preprint (which was not certified by peer review) is the author/funder, who has granted bioRxiv a license to display the preprint in perpetuity. It is made available under aCC-BY 4.0 International license.

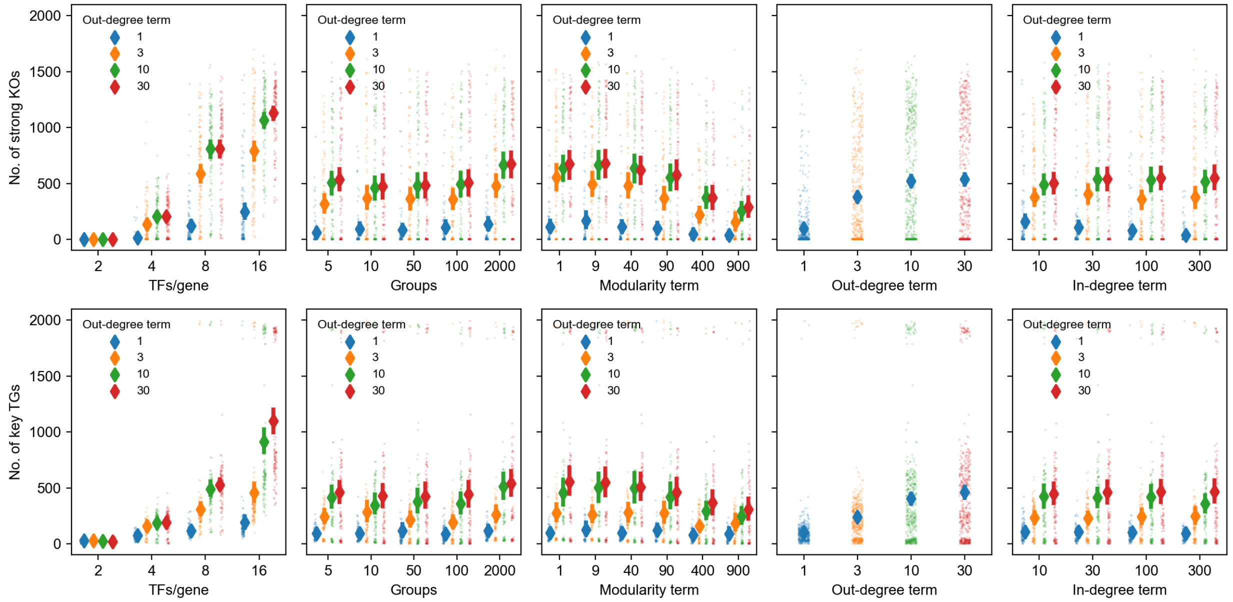

Figure S6: **No interaction between out-degree term and other network generating parameters**. Same as Fig. 4, but with additional stratification by the out-degree term δ_{out}. There is no obvious visual evidence for interactions between the parameters.

bioRxiv preprint doi: https://doi.org/10.1101/2024.07.04.602130; this version posted October 22, 2024. The copyright holder for this preprint (which was not certified by peer review) is the author/funder, who has granted bioRxiv a license to display the preprint in perpetuity. It is made available under aCC-BY 4.0 International license.

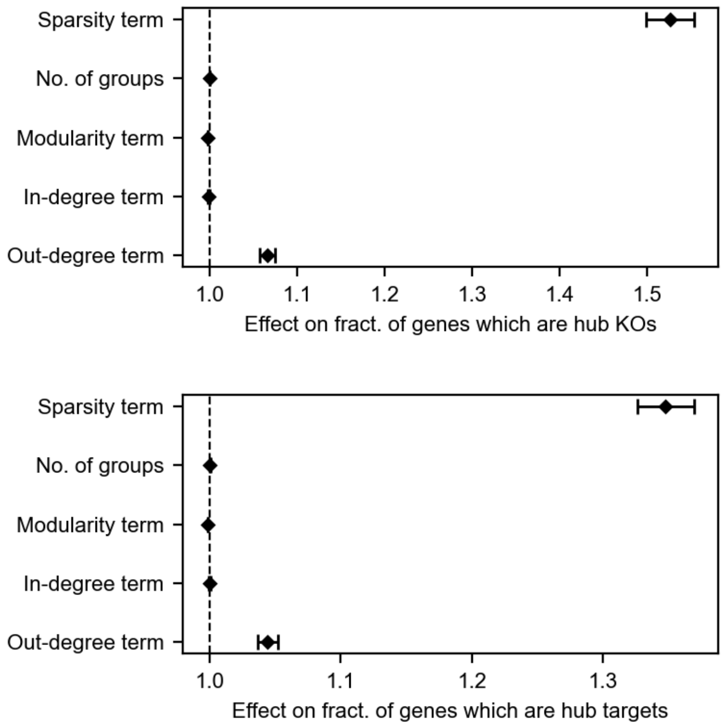

Figure S7: **Summary of regression models for effects of network parameters on perturbations**. Coefficients from regressing the logit-transformed fraction of genes which are hub knockouts (top) or target genes (bottom) on network generating parameters. Errorbars denote 95% confidence intervals for the regression coefficients. Model summaries can be found in Tables **S1** and **S2**.

bioRxiv preprint doi: https://doi.org/10.1101/2024.07.04.602130; this version posted October 22, 2024. The copyright holder for this preprint (which was not certified by peer review) is the author/funder, who has granted bioRxiv a license to display the preprint in perpetuity. It is made available under aCC-BY 4.0 International license.

**Dep. Variable:**	logit(pct_ko)	**R-squared:**	0.587
**Model:**	OLS	**Adj. R-squared:**	0.586
**Method:**	Least Squares	**F-statistic:**	543.6
**Date:**	–	**Prob (F-statistic):**	0.00
**Time:**	–	**Log-Likelihood:**	-4167.4
**No. Observations:**	1920	**AIC:**	8347.
**Df Residuals:**	1914	**BIC:**	8380.
**Df Model:**	5		
**Covariance Type:**	nonrobust		

	**coef**	**std err**	**t**	**P> |t|**	**[0.025**	**0.975]**
**const**	-6.9629	0.115	-60.458	0.000	-7.189	-6.737
**r**	0.4229	0.009	46.789	0.000	0.405	0.441
**k_adj**	0.0004	6.18e-05	6.857	0.000	0.000	0.001
**w**	-0.0023	0.000	-15.724	0.000	-0.003	-0.002
**delta_in**	-0.0012	0.000	-2.801	0.005	-0.002	-0.000
**delta_out**	0.0637	0.004	15.061	0.000	0.055	0.072

Table S1: Summary of regression results (fraction of genes which are hub knockouts).

bioRxiv preprint doi: https://doi.org/10.1101/2024.07.04.602130; this version posted October 22, 2024. The copyright holder for this preprint (which was not certified by peer review) is the author/funder, who has granted bioRxiv a license to display the preprint in perpetuity. It is made available under aCC-BY 4.0 International license.

**Dep. Variable:**	logit(pct_tg)	**R-squared:**	0.461
**Model:**	OLS	**Adj. R-squared:**	0.460
**Method:**	Least Squares	**F-statistic:**	327.7
**Date:**	–	**Prob (F-statistic):**	6.30e-254
**Time:**	–	**Log-Likelihood:**	-3973.5
**No. Observations:**	1920	**AIC:**	7959.
**Df Residuals:**	1914	**BIC:**	7992.
**Df Model:**	5		
**Covariance Type:**	nonrobust		

	**coef**	**std err**	**t**	**P> |t|**	**[0.025**	**0.975]**
**const**	-4.7854	0.104	-45.966	0.000	-4.990	-4.581
**r**	0.2983	0.008	36.514	0.000	0.282	0.314
**k_adj**	0.0003	5.59e-05	5.686	0.000	0.000	0.000
**w**	-0.0016	0.000	-12.002	0.000	-0.002	-0.001
**delta_in**	-0.0002	0.000	-0.438	0.661	-0.001	0.001
**delta_out**	0.0433	0.004	11.345	0.000	0.036	0.051

Table S2: Summary of regression results (fraction of genes which are hub target genes).

bioRxiv preprint doi: https://doi.org/10.1101/2024.07.04.602130; this version posted October 22, 2024. The copyright holder for this preprint (which was not certified by peer review) is the author/funder, who has granted bioRxiv a license to display the preprint in perpetuity. It is made available under aCC-BY 4.0 International license.

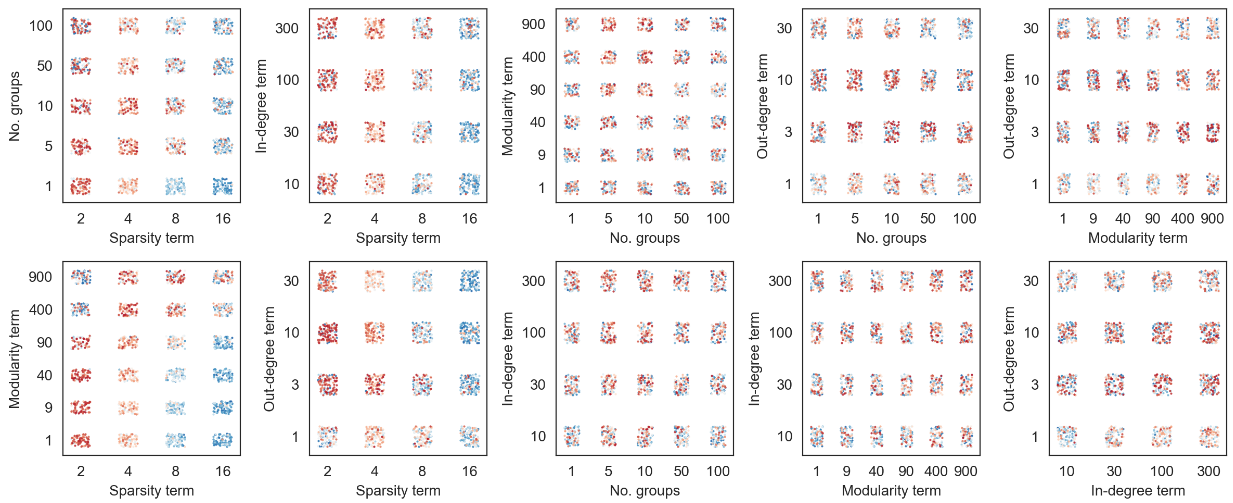

Figure S8: No interaction between network generating parameters and fit to experimental data. As in Fig. 5C-E, we show the relationship between pairs of network generating parameters and goodness of fit to the cumulative distribution of perturbation effects from experimental Perturb-seq data. Each GRN (one point in every subpanel) is colored by its ranked fit to data: the synthetic GRNs are ranked separately by Kolmogorov-Smirnov p-value for incoming and outgoing perturbation effects, then the sum of these two ranks is used to produce an overall ranking. Intense red color indicates better ranked fit to data, and intense blue color indicates a worse ranking.

bioRxiv preprint doi: https://doi.org/10.1101/2024.07.04.602130; this version posted October 22, 2024. The copyright holder for this preprint (which was not certified by peer review) is the author/funder, who has granted bioRxiv a license to display the preprint in perpetuity. It is made available under aCC-BY 4.0 International license.

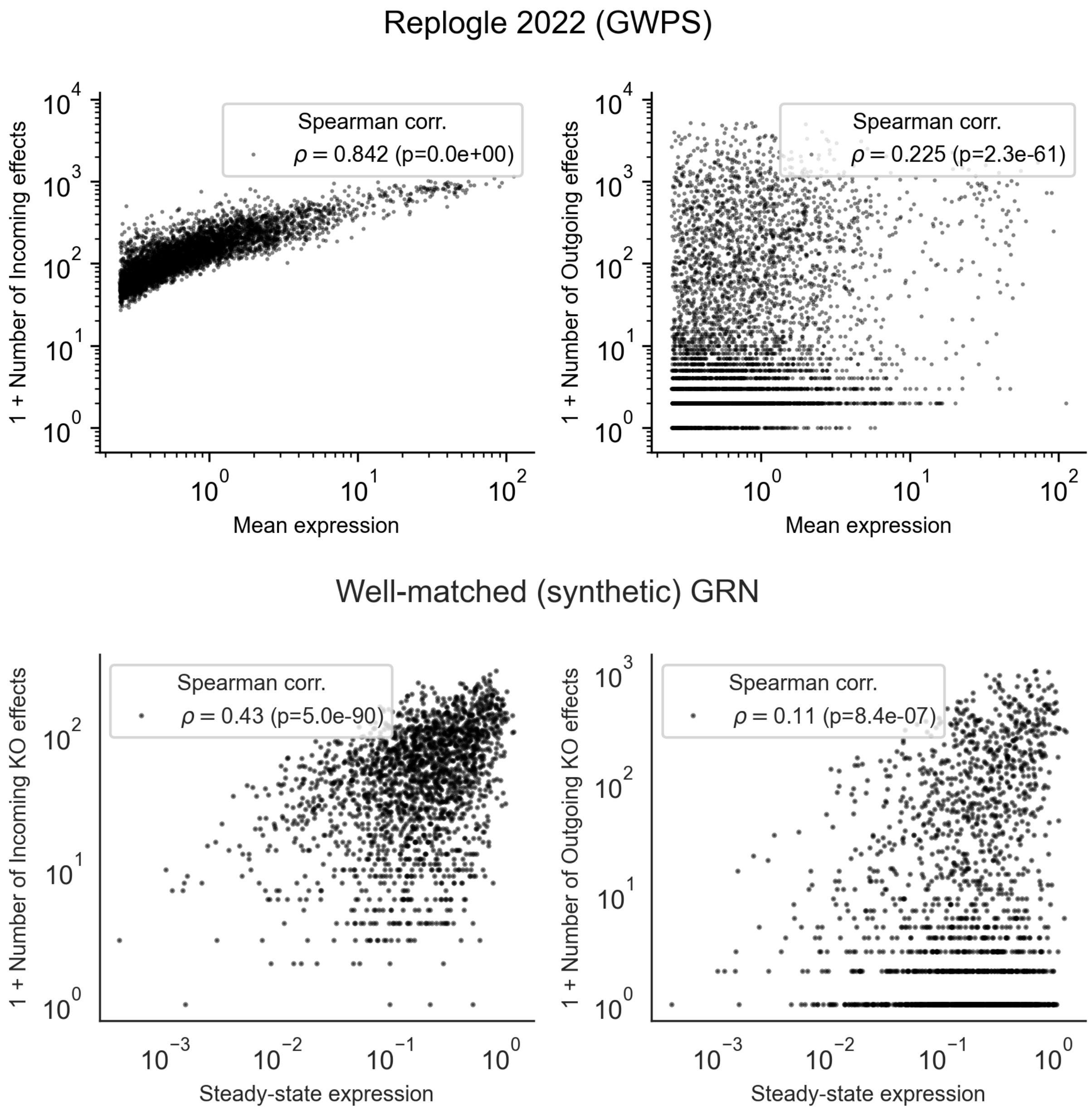

Figure S9: **Baseline expression influences the number of outgoing and incoming perturbation effects**. In the subsetted data from Replogle et. al. [9], and in the focal GRN from Fig. 6, we show the relationship between mean expression (in control cells in the experimental data; top panels) or steady-state expression (in the synthetic GRN; bottom panels) and the number of incoming (left) or outgoing (right) perturbation effects. Baseline expression relates to both of these quantities in both data sets: the relationships are stronger in the experimental data in part due to limits on detection power (especially important for incoming effects).

bioRxiv preprint doi: https://doi.org/10.1101/2024.07.04.602130; this version posted October 22, 2024. The copyright holder for this preprint (which was not certified by peer review) is the author/funder, who has granted bioRxiv a license to display the preprint in perpetuity. It is made available under aCC-BY 4.0 International license.

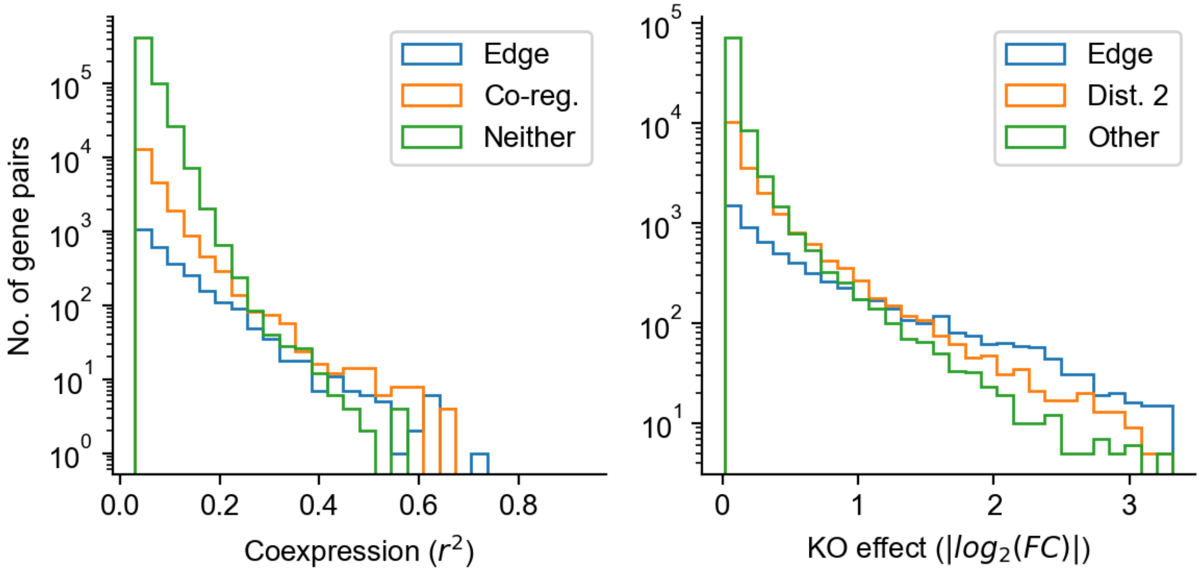

Figure S10: **Coexpression is more often due to coregulation than edges**. In the focal GRN from Fig. 6, we show a histogram of coexpression values split by whether pairs of genes share an edge ("A regulates B, or B regulates A", share a regulator ("A and B are coregulated"), or have another relationship (left panel). Similarly, for perturbation effects, we show the distribution split by whether pairs of genes share an edge ("A regulates B"), a path of distance 2 ("A indirectly regulates B"), or another relationship (right panel). At nearly all levels of coexpression, coregulation is more common than direct regulation. Meanwhile, direct regulation is more common than indirect regulation for the largest perturbation effects – note that the range of KO effects is clipped as in **Fig. 6**.

bioRxiv preprint doi: https://doi.org/10.1101/2024.07.04.602130; this version posted October 22, 2024. The copyright holder for this preprint (which was not certified by peer review) is the author/funder, who has granted bioRxiv a license to display the preprint in perpetuity. It is made available under aCC-BY 4.0 International license.

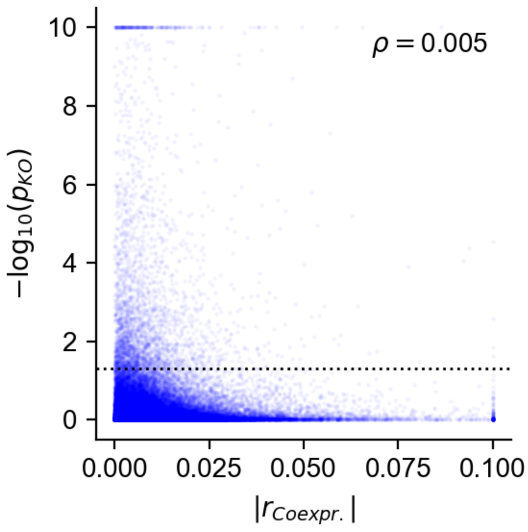

Figure S11: Baseline coexpression and perturbation effects are uncorrelated in Perturb-seq data. Same as **Fig. 6E**, using data from our analysis subset of Replogle *et. al.* 2022 [9]. Gene co-expression (x-axis) is the unsigned Pearson correlation between normalized single-cell gene expression data from unperturbed cells (clipped at $|r| = 0.1$). Perturbation effects (y-axis) are pairwise log-transformed Anderson-Darling p-values for differences in gene expression distribution between perturbed and unperturbed states (clipped at $-\log_{10}(p) = 10$). Rank correlation (Spearman's ρ) is computed on the transformed but not clipped values of these two statistics.

bioRxiv preprint doi: https://doi.org/10.1101/2024.07.04.602130; this version posted October 22, 2024. The copyright holder for this preprint (which was not certified by peer review) is the author/funder, who has granted bioRxiv a license to display the preprint in perpetuity. It is made available under aCC-BY 4.0 International license.

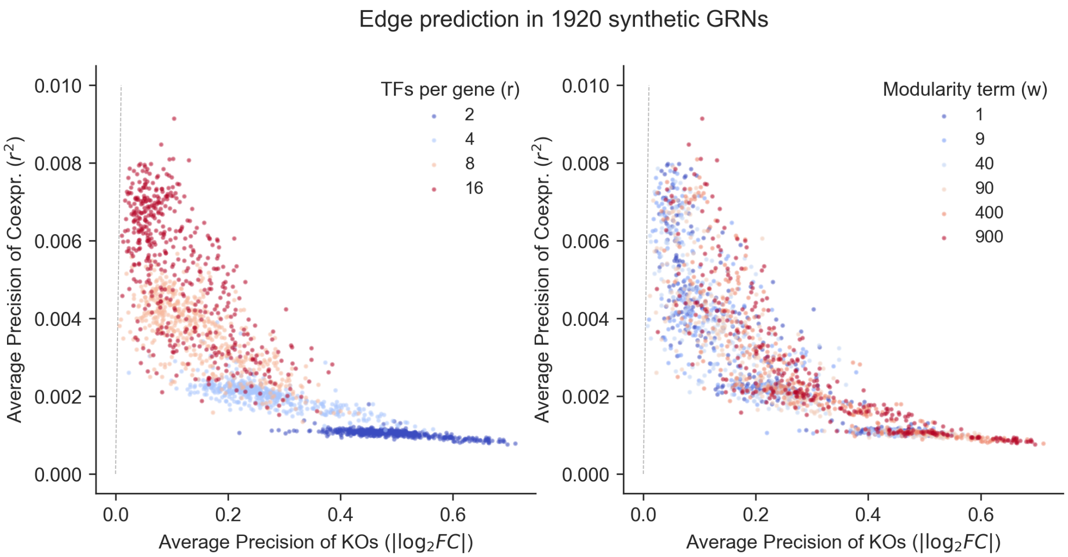

Figure S12: Graph properties inform the extent to which perturbation effects more strongly enrich for edges than co-expression. Performance of perturbation effects (x-axis, $|\log_2 \mathrm{FC}|$) and co-expression (y-axis, r^2 between genes) in identifying edge. As a summary performance measure, we compute the average precision (AP) score during binary classification of pairs of genes as being connected an edge (ignoring direction). All 1,920 networks in the study are shown in both panels, and colored by parameters of interest: the sparsity parameter (r, left) and the group affinity parameter (w, right). Across networks, sparsity and modularity degrade the performance of coexpression values, but enhance the performance of perturbation effects; but in every network, perturbation effects outperform coexpression (all points are below the dashed grey line, $y = x$).

bioRxiv preprint doi: https://doi.org/10.1101/2024.07.04.602130; this version posted October 22, 2024. The copyright holder for this preprint (which was not certified by peer review) is the author/funder, who has granted bioRxiv a license to display the preprint in perpetuity. It is made available under aCC-BY 4.0 International license.

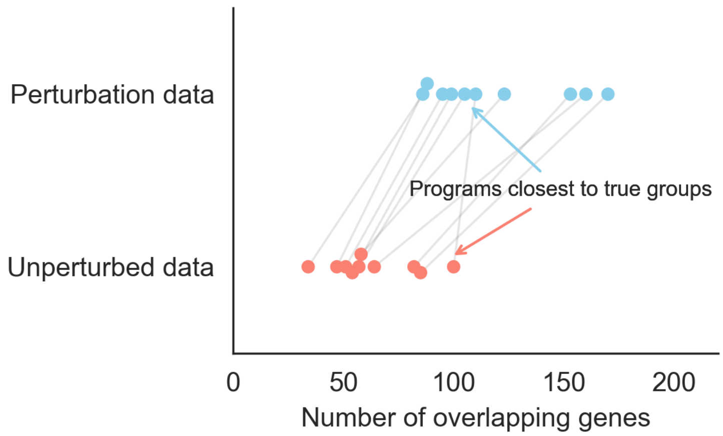

Figure S13: True groups in the synthetic GRN are represented among gene programs. In the focal GRN from Fig. 7, we show the overlap between each of the true groups ($k = 10$, shown as points in each of the bins on the y-axis) and its closest matching program (maximum overlap across all 50 gene sets, values shown on the x-axis). Points corresponding to the same true group are connected with a line spanning across y-axis bins. There is similar representation of all of the groups among the learned gene programs, regardless of input data type.

bioRxiv preprint doi: https://doi.org/10.1101/2024.07.04.602130; this version posted October 22, 2024. The copyright holder for this preprint (which was not certified by peer review) is the author/funder, who has granted bioRxiv a license to display the preprint in perpetuity. It is made available under aCC-BY 4.0 International license.

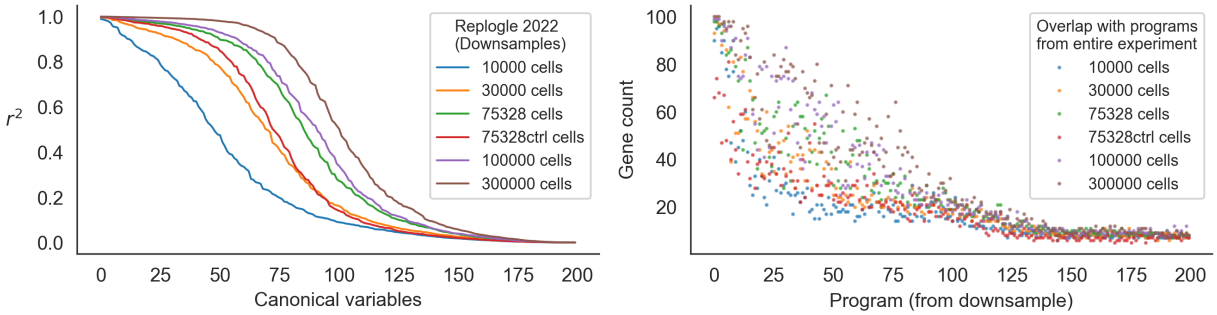

Figure S14: Program replication depends on the number of cells. Same as Fig 7C,D – instead of taking downsamples of unperturbed cells from Replogle *et. al.*, 2022, we here downsample the entire experiment to various study sizes. Here, the "entire experiment" is the normalized expression measurements of 5,247 genes in 932,593 control and intervened-upon cells which received one of the 5,247 perturbations in our analysis subset (**Methods**). We compare singular vectors (left) and programs (right) from the resulting downsamples of the entire experiment, as well as the subsets from Fig 7C,D. We note that the 75,328 control cells replicate the programs from the entire dataset comparably to 30,000 cells from the entire experiment.

bioRxiv preprint doi: https://doi.org/10.1101/2024.07.04.602130; this version posted October 22, 2024. The copyright holder for this preprint (which was not certified by peer review) is the author/funder, who has granted bioRxiv a license to display the preprint in perpetuity. It is made available under aCC-BY 4.0 International license.

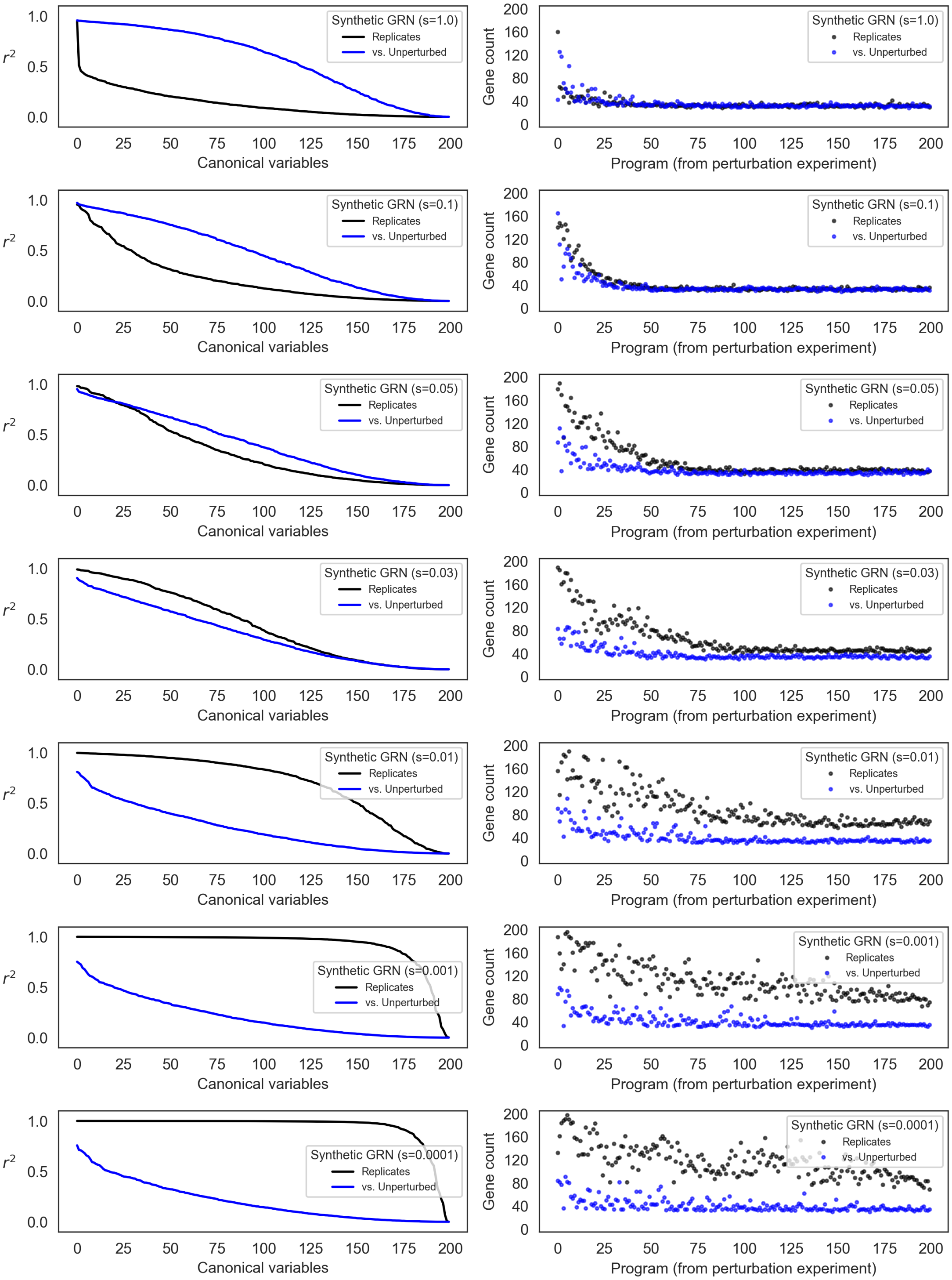

Figure S15: Program replication depends on the magnitude of intrinsic noise. Same as Fig 7A,B for different levels of noise. We repeat CCA and analysis of gene programs as in Fig 7 (see **Methods**), varying the level of intrinsic noise (s). At low levels of noise (small s), replicates from perturbed conditions are much more similar to one another than to the unperturbed data. At high levels of noise (large s), the perturbed data are more similar by canonical correlation to the unperturbed data than to the replicate perturbed data; but programs derived from each of the singular vectors are equivalently reproducible across conditions.
